# A Friendly Complexing Agent for Spectrophotometric Determination of Total Iron

**DOI:** 10.3390/molecules26113071

**Published:** 2021-05-21

**Authors:** Valeria M. Nurchi, Rosita Cappai, Nadia Spano, Gavino Sanna

**Affiliations:** 1 Dipartimento di Scienze della Vita e dell’Ambiente, Università di Cagliari, Cittadella Universitaria, 09042 Cagliari, Italy; cappai@unica.it; 2 Dipartimento di Chimica e Farmacia, University of Sassari, Via Vienna 2, 07100 Sassari, Italy; nspano@uniss.it

**Keywords:** iron, total iron, desferal, desferrioxamine B, spectrophotometry

## Abstract

Iron, one of the most common metals in the environment, plays a fundamental role in many biological as well as biogeochemical processes, which determine its availability in different oxidation states. Its relevance in environmental and industrial chemistry, human physiology, and many other fields has made it necessary to develop and optimize analysis techniques for accurate determination. Spectrophotometric methods are the most frequently applied in the analytical determination of iron in real samples. Taking advantage of the fact that desferrioxamine B, a trihydroxamic acid used since the 1970s in chelation therapy for iron overload treatment, forms a single stable 1:1 complex with iron in whichever oxidation state it can be found, a smart spectrophotometric method for the analytical determination of iron concentration was developed. In particular, the full compliance with the Lambert-Beer law, the range of iron concentration, the influence of pH, and the interference of other metal ions have been taken into account. The proposed method was validated in terms of LoD, LoQ, linearity, precision, and trueness, and has been applied for total iron determination in natural water certified material and in biological reference materials such as control human urine and control serum.

## 1. Introduction

Iron is an extremely widespread metal of high relevance in environmental, industrial and biological contexts. Therefore, the development of reliable analytical methods devoted to measuring its total concentration is of primary importance. Atomic absorption spectroscopy (AAS), ion chromatography (IC), inductively coupled plasma (ICP-AES and ICP-MS), controlled-potential methods, and ultraviolet-visible spectrophotometry (UV-vis) are the most used techniques.

The design and development of spectrophotometric methods for quantitative detection has gained increasing interest among analytical chemists due to their high selectivity, sensitivity, cost-effectiveness, simplicity, and low detection limit. Factually, different reagents, each one characteristic for a single iron oxidation state, have been used for (Table 1 and Table 2) [1,2,3]. Despite the high sensitivity allowed by the use of these reagents, the methods require troublesome procedures for total iron determination.

**Figure 1:**
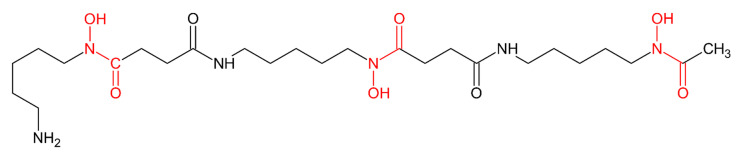
Molecular structure of DFO highlighting the three iron chelating hydroxamic groups.

We reported in a chapter on iron chelators [7] the history of the serendipitous discovery of desferrioxamine B mesylate (desferal, DFO) and of its introduction in clinical practice. It is a trihydroxamic acid (Figure 1) of natural origin used in clinical medicine for scavenging Fe^3+^ in iron overload pathologies, mainly in β-thalassemia patients.

DFO has been used for many years in our group for the standardization of Fe^3+^ solutions employed in complex formation equilibrium studies [8], and thanks to the awareness of the properties of this ligand, the use of DFO immobilized in solid as support for a sensor and as sorbent for iron was proposed [9,10,11,12]. Since DFO forms an extremely stable intensely red colored 1:1 complex with Fe^3+^ in a wide pH range, and the same complex is immediately formed in air even starting from a Fe^2+^ solution, here we present its analytical applications in the spectrophotometric determination of total iron, and a thorough evaluation of its pros and cons.

## 2. Results and Discussion

### 2.1. Spectrophotometric Analysis and Requisites of Colorimetric Reagents

The analytical determination of an analyte by UV-vis spectrophotometry is based on the Lambert-Beer law Equation (1):

A = ε b C,(1)

A being the measured absorbance, ε the absorptivity, C the concentration of analyte, and b the optical path length. Knowing ε and b, the measure of the absorbance of unknown samples allows the estimation of the analyte concentration. The determination of absorptivity ε from a calibration plot gives additional evidence of any deviation from linearity of the absorbance vs. concentration plot. The used concentrations should allow to measure absorbance values in the range 0.1 ÷ 2. The ε value and the used path length b determine the range of concentration of the analytical determination. When the analyte is a transition metal ion, the colored aquo ion is characterized by ε values ~100 M^−1^ cm^−1^, which allow to evaluate concentrations 10^−2^ M using a 1 cm path length. In the case of iron, a concentration 10^−2^ M corresponds to 558 mg/L, too high for any analytical relevance. Thus, for determining lower iron concentrations, it is necessary to form iron complexes characterized by higher absorptivity values with a proper reagent. The used ligand, a colorimetric reagent, must possess several characteristic features to be considered of analytical interest for assessing spectrophotometric methods. In particular:

(1) Formation of a single complex of a definite stoichiometry, stable in a wide pH range;
(2) High stability of the formed complex;
(3) Fast reaction of complex formation;
(4) Selectivity toward the target metal ion;
(5) High values of absorptivity ε.

### 2.2. Main Features of the Fe^3+^-DFO Interactions

**Figure 2:**
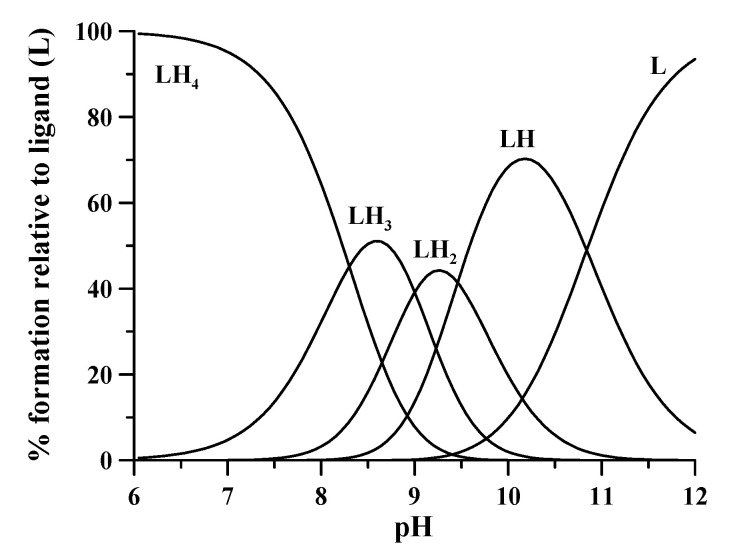
Speciation plot of differently protonated forms of DFO calculated with the HySS program [14]. L, in complex formation equilibrium studies, represents the completely deprotonated form of ligand. Charges are omitted for simplicity.

The main features of DFO, based on literature results, are reported to appreciate its compliance with the requisites of a colorimetric reagent. The value of the octanol/water partition coefficient (log *p* = 0.614) is indicative of the high hydrophilic character of DFO, and so of water solubility > 20% at 20 °C [13]. The four protonation constants at 25 °C and 0.2 M ionic strength are log K_1_ = 10.84, log K_2_ = 9.46, log K_3_ = 9.00, and log K_4_ = 8.30, the first one being attributed to the terminal amino group and the remaining three to the hydroxamic groups [7]. Figure 2 shows the related speciation plot.

**Figure 3:**
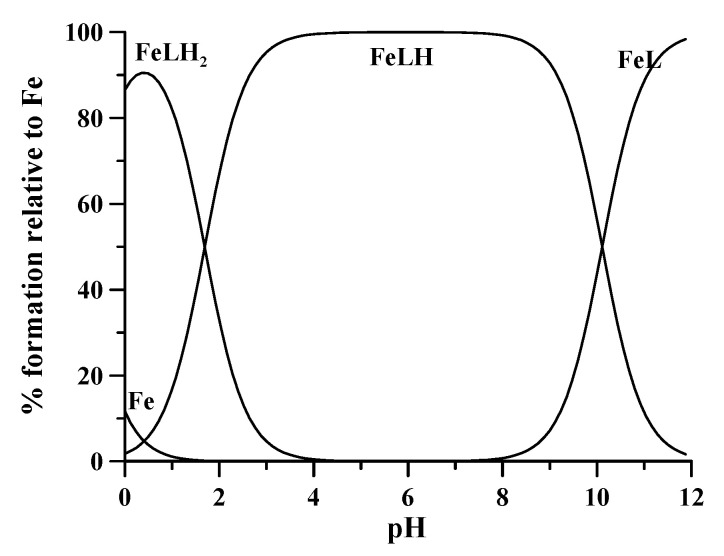
Speciation plot of different Fe^3+^-DFO complexes, calculated with the HySS program at Fe^3+^ concentration of 4.5 × 10^−5^ M and DFO concentration of 1.6 × 10^−3^ M. Charges are omitted for simplicity.

DFO forms three differently protonated 1:1 complexes with Fe^3+^: [FeLH_2_]^2+^, [FeLH]^+^, and FeL, characterized by complex formation constants log β_112_ 42.4, log β_111_ 41.01, and log β_110_ 30.4, respectively [15]. The speciation plot in Figure 3 shows that at extremely acidic conditions, pH values between 0 and 1, the biprotonated complex is almost completely formed, in which iron is coordinated by two hydroxamic groups of DFO, the third group being still protonated, as well as the terminal amino group. The formation of the FeLH complex, in which the iron ion is octahedrally coordinated by all the three hydroxamic groups of DFO starts at pH about 1. This monoprotonated complex is formed at a percentage > 99.2 at pH 3.5 and it loses the proton on the terminal amino group with pK 10.61, very close to the value of 10.84 in the free ligand. This implies that the terminal amine does not play any role in iron coordination.

**Figure 4:**
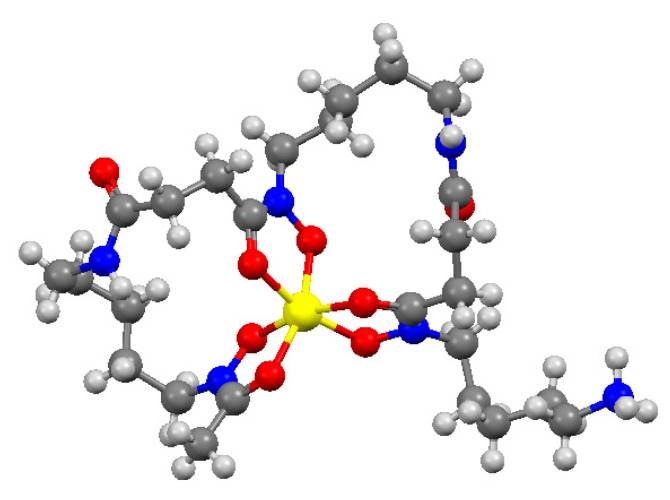
Crystal structure of the monoprotonated Fe^3+^-DFO complex (H white; O red; N blue; C grey; Fe yellow). Coordinates obtained from Cambridge Structural Database, CSD entry code OFUYET, image made by Mercury 3.5.

The crystal structure of the [FeLH]^+^ complex (Figure 4), reported in 2001 by the Crumbliss group [16], is described by two closed loops and an open chain with the protonated amine that points out from the chelate rings.

This compact structure around the Fe^3+^ metal ion determines the high stability of the complex, and the extremely low redox potential −475 mV at pH 7.5 [17], which completely stabilizes the ferric oxidation state. This peculiar characteristic of DFO allows the determination of total iron concentration independently from the starting oxidation state. The redox potential −475 mV ensures that any amount of Fe^2+^ is completely oxidized to form the Fe^3+^-DFO complex. Actually, DFO is a perfect candidate to be a ligand for the spectrophotometric determination of total iron. First, it forms only 1:1 differently protonated complexes with extremely high stability and the monoprotonated complex is stable as a unique complex existing in the 3.5 ÷ 8 pH range. In addition, literature findings account for very fast kinetic of formation of the [FeLH]+ complex from DFO and Fe^3+^ at pH values > 3 [18,19,20]. Conversely, 5 ÷ 10 min are required for the protonation of the [FeLH]+ complex in an acid environment to obtain the [FeLH_2_]^2+^ complex, where iron is coordinated by only two hydroxamate groups. Hence, literature evidences support the accomplishment of the first three features for the eligibility of DFO to be a reliable complexing agent.

### 2.3. Calibration Plot

**Figure 5:**
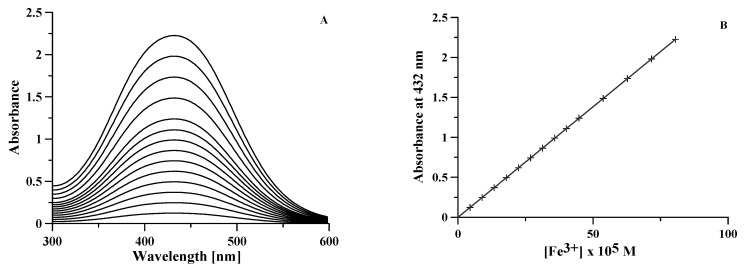
(**A**) Spectra of 14 Fe^3+^-DFO solutions with [DFO] = 1.6 × 10^−3^ M and [Fe^3+^] ranging from 4.5 × 10^−5^ M to 8 × 10^−4^ M. (**B**) Calibration plot of the absorbance at 432 nm vs. iron concentration for each of the 14 solutions; regression line calculated for a straight line through the origin is reported as a continuous line.

To check the spectral behavior of the system, we recorded the spectra of 14 solutions of Fe^3+^ at variable concentrations from 4.5 × 10^−5^ M to 8 × 10^−4^ M, whose absorbance values in the maximum range from 0.1 to 2 absorbance units. These solutions were accurately prepared, starting from a commercial ICP standard solution of Fe^3+^ with a declared concentration of 1000 mg/L from Fe(NO_3_)_3_ (corresponding to an iron concentration 1.7907 × 10^−2^ M) with an excess of nitric acid 0.3 M, and density 1.015 g/mL. The concentrations of the 14 solutions were extremely accurate, each one being prepared by picking up increasing amounts of Fe^3+^ stock solution determined by weighting with a four-digit balance, after introduction in a 25 mL volumetric flask. Then, 5 mL of DFO solution 0.008 M was added to the flask, so that in the more concentrated solution the DFO concentration was twice that of iron. Moreover, a proper volume of NaOH 0.1 M was added to neutralize either the amount of H^+^ introduced with the iron standard solution, or that released from DFO during complexation. The volumetric flask was made up to the mark, and the final volumes were checked by weighting. The pH of these 14 solutions were measured and the spectra recorded in the 300 ÷ 600 nm spectral range. The pH was almost constant between 6.8 and 7.1, in the pH range of the existence of monoprotonated [FeLH]^+^ complex (Figure 3). The recorded spectra and the calibration curve are shown in Figure 5. The data perfectly conform to the Lambert-Beer law in the used concentration range with an absorptivity value 2764.8(1) M^−1^ cm^−1^ at 432 nm.

### 2.4. Available pH Range

**Figure 6:**
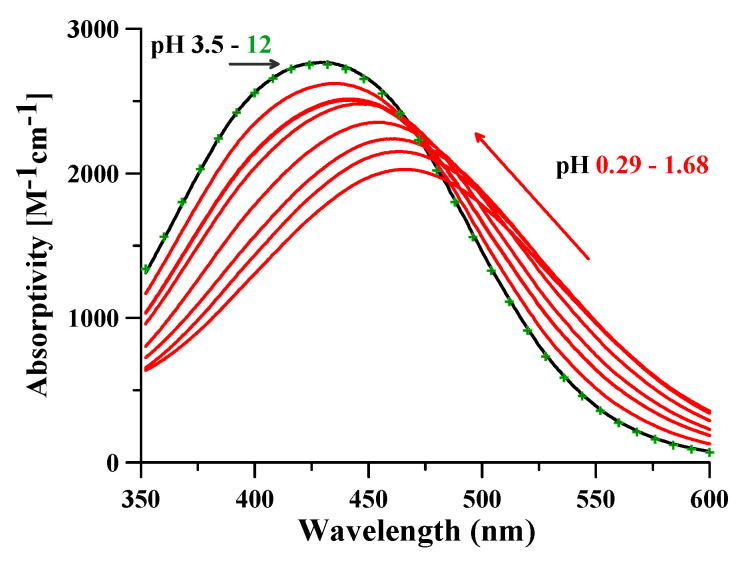
Absorptivity spectra of the Fe^3+^-DFO solutions with [Fe^3+^] = 4.0 × 10^−4^ M and 1:2 metal:ligand ratio at increasing pH: from pH 0.29 to 1.68 in red, pH 3.5 in black, and pH 12 in green symbols +.

To evaluate the spectral contribution of the differently protonated forms other than the monoprotonated complex [FeLH]^+^ (Figure 3), the spectra of a set of solutions Fe^3+^-DFO were collected at constant iron and DFO concentrations (1:2 metal:ligand molar ratio with [Fe^3+^] = 4.0 × 10^−4^ M), and at pH variable from 0.29 to 12. The 12 spectra obtained in the pH interval 3.5 ÷ 12 completely overlap, giving evidence that the deprotonation of amine group is spectrally silent. Conversely, meaningful changes were observed in the UV-vis spectra recorded at pH < 3.5. A bathochromic shift joined to a hypochromic effect was observed at lowering pH, attributable to the progressive formation of the [FeLH_2_]^2+^ complex. Figure 6 summarizes the above observations. Therefore, the 1:1 complexes [FeLH]+ and FeL, very stable in the pH interval 3.5 ÷ 12, present the same spectral feature, all the spectra in this range being completely indistinguishable. This represents a great advantage of the proposed method. In fact, once any spectrophotometric measurement below pH 3.5 is avoided, no buffering is required in the experimental procedure in the range of 3.5 ÷ 12.

### 2.5. Effects of Other Metal Ions

Metal ions other than iron present in the solution could interfere (i) by competing with iron for DFO complexation, depending both on the ratio between the complex formation constants of the metal ion and iron, and on the ratio of their concentrations, or (ii) by forming an absorbing complex with the DFO in excess, affecting the spectra of the Fe^3+^-DFO complex. In regards to the direct competition between interfering ions and Fe^3+^ towards complexation with DFO, Table 3 reports literature values of the stability constants of DFO complexes with trivalent metal ions such as Al^3+^, In^3+^, and Ga^3+^, as well as divalent ones such as Cu^2+^, Ni^2+^, and Zn^2+^. In addition, the pM values for each considered metal ion are reported, where pM is the negative logarithm of the concentration of the free metal ion in solution, calculated for total [ligand] = 10^−5^ M and total [metal] = 10^−6^ M at pH 7.4 [21].

The data reported in Table 3 show that DFO forms complexes of high stability with all trivalent metal ions, of medium stability with Cu^2+^, and of low stability with the remaining bivalent metal ions. In order to clarify if the presence of a metal ion can prevent the complete complexation of iron by DFO, the effects of Al^3+^ and Ga^3+^, the two metal ions that form the stronger complexes, were investigated by using the HySS program [14]. The input concentrations for the program calculation were 5 × 10^−4^ M for iron and 1.6 × 10^−3^ M for DFO. Instead, three different concentrations for Al^3+^ and Ga^3+^ ions were used: 5 × 10^−4^ M i.e., equal to that of iron, 5 × 10^−3^ M, 10 times in excess, and 5 × 10^−2^ M, 100 times in excess. Table 4 presents the calculated concentrations of [FeLH]^+^, the form stable in the pH range 3.5 ÷ 8, at variable concentrations of the perturbing metal ions.

Only Ga^3+^ prevents in a minimal amount the complete formation of the [FeLH]^+^ complex, leaving less than 0.03% of total iron uncomplexed when 10 times in excess and less than 0.4% when 100 times in excess. HySS calculations confirm the very high selectivity of DFO for iron, whose complex formation is also almost unaffected by large excess of different metal ions.

Cu^2+^ and Ni^2+^ ions were chosen to evaluate the capability of other ions to form colored complexes with the excess of DFO. Although Van Reyk and Dean [25] reported the possibility that DFO reduces Cu^2+^, the paper by Farkas et al. [24] does not give any evidence of this and, conversely, presents EPR evidence that copper is on +2 oxidation state in the Cu-DFO complexes. Therefore, the absorbance of the two systems Cu^2+^-DFO and Ni^2+^-DFO at pH 7 was measured at concentrations 5 × 10^−4^ M of the metal ions and 1.6 × 10^−3^ M of the ligand. Based on stability constants in Table 3 and on speciation plot at the above concentrations, the species at pH 7 are [CuLH_2_]^+^ and [NiLH_2_]^+^, respectively. In particular, they present absorbance values at 432 nm of 0.030 and 0.015, respectively, whereas the absorbance value at the same wavelength of the [FeLH] ^+^ complex is 1.38. Hence, the interference of both these bivalent metal ions on the analyte are 2.2% and 1.1%, respectively.

### 2.6. Determination of Total Iron

The most used analytical methods in the determination of total iron is the complexation of Fe^2+^ by 1,10-phenantroline or 2,2′-bipyridine, after reduction of all the present Fe^3+^. Goodwin and Whitten [26] report the ability of DFO to react with ferrous iron, resulting in the oxidized Fe^3+^-DFO complex. This instantaneous oxidation takes place in the presence of oxygen, despite the presence of reducing agents as ascorbic acid and sodium sulphite in solution. Successively, Yegorov et al. [27] proposed a method for the simultaneous determination of both forms of iron using DFO. The applicability of the method to the determination of total iron in solution has hence been tested. DFO complexes from solutions of either Fe^2+^ or Fe^3+^ 5 × 10^−4^ M or of a mixture [Fe^2+^] = [Fe^3+^] = 2.5 × 10^−4^ M, and DFO 1.6 × 10^−3^ M at pH 7 provided the same UV-vis absorption spectra. No significant differences were observed on further spectra collected at pH values ranging between 3.5 and 8.

Even if on one side, both Fe^2+^-1,10-phenantroline and Fe^2+^-2,2′-bipyridine complexes show molar absorptivity (ε of 10,281 M^−1^ cm^−1^ at 510 nm and of 8558.1 M^−1^ cm^−1^ at 520 nm, respectively) higher than that exhibited by Fe^3+^-DFO complex (ε of 2764.8 M^−1^ cm^−1^ at 432 nm), on the other side, the complexity of the method affecting its precision and the long time required for the reduction are the key elements to prefer a simpler DFO method for total iron determination in matrices of a different nature.

### 2.7. Validation: LoD and LoQ

LoD was calculated according the method proposed by Currie [28], largely used for its recognized capability to minimize both type 1 and type 2 decision errors, as Equation (2):

(2) $$\mathrm{LoD}=\frac{3\;\cdot {\;\sigma }_{b}}{b}$$

σ_b_ being the standard deviation of a large number of blank measurements and b the slope of the calibration curve obtained in a range of analyte concentrations as close as possible to the tentative value of LoD. Specifically, the absorbance at 432 nm of 25 independent blanks was measured, leading to a mean value 0.00016 and a related standard deviation 0.00013.

Hence, three calibration curves in the range of Fe^3+^ concentration between 0.2 and 0.6 mg/L were prepared and a fixed excess of DFO was added to the standard solution of Fe^3+^ ion. The relevant equations of the calibration curves are y = 0.058(1)x + 0.001(1), y = 0.0428(8)x + 0.0005(6) and y = 0.049(1)x − 0.0002(2), respectively. The mean value of these three slopes corresponds to the slope previously reported for the calibration plot in Figure 5. The average value of the LoD, calculated from each of the three calibration curves, is 0.008 mg/L, and the LoQ, calculated as 3.3 times the LoD value, is 0.026 mg/L.

### 2.8. Precision

The precision of the proposed method was evaluated in terms of both repeatability and intermediate precision. Repeatability was measured by 20 consecutive measurements of maximum absorbance at 432 nm of Fe^3+^-DFO complex at iron concentrations 4.5 × 10^−5^ M = 2.51 mg/L (i.e., roughly 100 times higher than the LoQ). The evaluation of the intermediate precision was accomplished measuring 10 times the maximum absorbance of Fe^3+^-DFO complex at the same experimental condition as above once a week for five consecutive weeks. Repeatability was always better than 0.8%, whereas the intermediate precision was 2.0%. The acceptability of the precision data was successfully verified in terms of Horwitz’s theory [29].

### 2.9. Trueness and Application to Reference Materials

Trueness was evaluated by analyzing three certified reference materials (CRM) of abiotic and biotic origin, containing an iron concentration between 93.44 μg/L and 905 μg/L. Table 5 reports for each CRM, the certified concentration of iron, the measured concentration, and the relevant per cent recovery.

Very good recoveries were found for all the CRM samples considered. For natural water, its great simplicity and the possibility to be used in both on-field and screening measurements offers large applicability of this method. As a matter of fact, iron is normally present in up to concentrations of 10 mg/L in groundwater, up to 0.7 mg /L in river water, and up to 0.3 mg/L in drinking water [30], i.e., for analyte concentrations high enough to provide direct, fast, and reliable measurements. The method was also successfully applied to biological fluids like urine and serum.

## 3. Materials and Methods

### 3.1. Chemicals

DFO was a Novartis product, whose purity was verified by potentiometric titration and NMR spectroscopy. NIST SRM 1643f Natural Water certified reference material, FeCl_3_, CuCl_2_, NiCl_2_, (NH_4_)2Fe(SO_4_)2·6H_2_O, HCl, NaOH, iron standard for ICP (1000 mg/L in Fe^3+^, from Fe(NO_3_)_3_ in HNO_3_ 0.3 M, d 1.015 g/mL) were Aldrich products used without purification. The ClinChek^®^ Control Human Urine—Level II, code 8848, and the ClinChek^®^ Control Blood Serum-Level IA, code 8880 were from Recipe (Munich, Germany). Carbonate-free 0.1 M NaOH solution was prepared according to Albert and Serjeant [31]. The metal ion standard solutions were prepared by dissolving the required amount of chloride salts in pure double distilled water and adding a stoichiometric amount of HCl to prevent hydrolysis. Cu^2+^ and Ni^2+^ solutions were standardized by EDTA titration.

### 3.2. Instrumentation

Potentiometric titrations were performed using a Metrohm 888 Titrando (Herisau, Switzerland), whereas the spectrophotometric measurements were accomplished using a Cary 60 UV-vis spectrophotometer (Agilent Technologies, Santa Clara, CA, USA) with 1 cm path length cell.

## 4. Conclusions

A simple and rapid spectrophotometric method to determine total iron concentration in different matrices was developed and validated. The method is based on the use of a complexing agent of natural origin, DFO, that forms complexes with Fe^3+^ 1:1 of high stability in a wide pH range. The extremely low redox potential of these complexes allows, in the presence of air, the oxidation and complexation of all Fe^2+^ eventually present in the solution. Although the molar absorptivity of the Fe^3+^-DFO complex is less than that of like 1,10-phenantroline or 2,2′-bipyridine, it allows a reliable measure of iron concentration well below the mg/L level. No meaningful interference by the most common trivalent and bivalent metal ions was observed in the operative pH range. The LoD of the method is 0.008 mg/L and the linearity exhibits full compliance with the Lambert-Beer law. Precision and trueness was ascertained on three certified materials, with always very good recovery. The method features, such as versatility of DFO, rapidity, low-cost, trueness, and selectivity, give strength to its eligibility in the determination of total iron in environmental and biological samples.

## Figures and Tables

**Table 1:** Most used ligands for Fe^2+^ determination and their spectral features.

	Spectral Info	Ref.
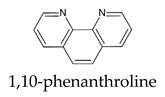	ε = 11,000 M^−1^cm^−1^ λmax = 512 nm	[4]
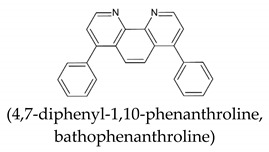	ε = 22,400 M^−1^cm^−1^ λmax = 533 nm	[5]
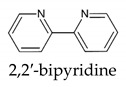	ε = 8700 M^−1^cm^−1^ λmax = 522 nm	[6]

**Table 2:** Most used ligands for Fe^3+^ determination and their spectral features.

	Spectral Info	Ref.
SCN^−^ thiocyanate	ε = 8530 M^−1^cm^−1^ λmax = 480 nm	[1]
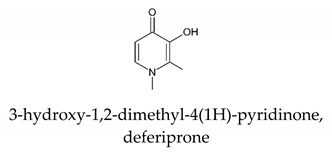	ε = 4400 M^−1^cm^−1^ λmax = 440 nm	[3]

**Table 3:** Complex formation constants of DFO complexes with Fe^3+^, Al^3+^, Ga^3+^, In^3+^, Cu^2+^, Ni^2+^, and Zn^2+^ and the related pM values. Charges are omitted for simplicity.

Metal Ion	[MLH_3_]	[MLH_2_]	[MLH]	[ML]	[M_2_LH]	pM	Ref.
Fe^3+^		42.4	41.01	30.4		26.5	[15]
Al^3+^		36.6	33.8	23.9		19.3	[22]
Ga^3+^			36.92	27.56		22.4	[23]
In^3+^		36.40	32.48	22.18		18.0	[17]
Cu^2+^	36.99	33.10	23.98	13.73	32.09	11.2	[24]
Ni^2+^	33.20	27.66	19.71	8.89		6.3	[18]
Zn^2+^	33.40	28.17	20.40	10.36		6.6	[18]

**Table 4:** Effects of increasing concentrations of Al^3+^ and Ga^3+^ ions on the formation of the absorbing complex [FeLH]^+^ at pH 6 and 7, calculated by the HySS program.

	Presence of Al^3+^		Presence of Ga^3+^	
Molar Concentration of Interfering Metal Ion	Molar Concentration × 10^4^			
	[FeLH]^+^ pH 6	[FeLH]^+^ pH 7	[FeLH]^+^ pH 6	[FeLH]^+^ pH 7
0	4.9998	4.9988	4.9998	4.9988
5 × 10^−4^	4.9998	4.9988	4.9998	4.9988
5 × 10^−3^	4.9998	4.9988	4.9983	4.9973
5 × 10^−2^	4.9997	4.9987	4.9818	4.9807

**Table 5:** Spectrophotometric determination (*n* = 5) with the proposed method of iron amount in different CRMs.

	Certified Iron Concentration (μg/L)	Measured Iron Concentration (μg/L)	Recovery (%)
NIST SRM 1643f Natural Water	93.4(0.8)	95(1)	102
ClinChek^®^ Control Human Urine Level II	222	215(2)	97
ClinChek^®^ Control Blood Serum Level I	905	935(7)	103

## Data Availability

All the data will be made available asking at nurchi@unica.it.

## Sample Availability

All the used compounds are commercially available.
